# P-1878. Intravenous Ganciclovir Administration Outcomes via an Outpatient Parenteral Antimicrobial Therapy (OPAT) Program

**DOI:** 10.1093/ofid/ofae631.2039

**Published:** 2025-01-29

**Authors:** Dhruv Patel, Ryan P Mynatt, Ashley Logan, Evelyn Villacorta Cari, Armaghan-E Rehman Mansoor

**Affiliations:** University of Kentucky, Ontario, California; University of Kentucky, Ontario, California; University of Kentucky HealthCare, Lexington, Kentucky; University of Kentucky, Ontario, California; University of Kentucky, Ontario, California

## Abstract

**Background:**

Intravenous (IV) ganciclovir is used in the management of herpesvirus infections including cytomegalovirus (CMV). Ganciclovir is usually administered in the inpatient setting given the need for close monitoring of hematologic and renal parameters. This study describes our experience with IV ganciclovir administration in immunocompromised hosts, via an OPAT program at a tertiary academic medical center. Ganciclovir was chosen as choice of therapy either due to concern for GI absorption, or provider preference.

**Methods:**

This is a retrospective, single-center review of patients discharged on IV ganciclovir via OPAT from August 2019 to December 2023. Patient demographics, underlying diagnoses, indication for ganciclovir, duration of therapy, treatment outcomes and adverse events were collected.

**Results:**

We included 18 patients whose mean age was 58.6 (SD±12.4) years, with ten (55.6%) being male. The most common underlying immunocompromising condition was receipt of a transplanted organ in 15 (83.3%) patients, including five and ten recipients of hearts and kidneys, respectively. The mean duration of therapy was 25 (SD±11) days. In our cohort, 13 were treated for CMV disease as follows; 11 patients with gastrointestinal involvement and two patients with encephalitis/retinitis. Four patients had isolated viremia and one patient was treated for Epstein Barr Virus encephalitis. A majority (13 patients, 72.2%) transitioned to valganciclovir on completion of parenteral therapy for secondary prophylaxis or to continue induction therapy.

The most common adverse event was worsening cytopenia in four (22.2%) patients, one of whom had baseline leukopenia. Neutropenia was documented in three patients, and one patient had thrombocytopenia. One (5.6%) patient developed acute kidney injury (AKI) through their treatment, requiring dose modification.

**Conclusion:**

Ganciclovir is a viable option for outpatient parenteral therapy in the treatment of CMV disease where an extended duration of IV therapy is required. In our cohort of 18 immunocompromised patients, only one had early discontinuation of therapy due to ganciclovir-related AKI. Close monitoring of labs through an established OPAT protocol is needed for successful completion of therapy.

**Disclosures:**

All Authors: No reported disclosures

